# Associations between *TNFSF4*, *TNFSF8* and *TNFSF15* and Behçet's disease but not VKH syndrome in Han Chinese

**DOI:** 10.18632/oncotarget.22064

**Published:** 2017-10-23

**Authors:** Yan Jiang, Ling Cheng, Xin Li, Wenke Zhou, Li Zhang

**Affiliations:** ^1^ The First Affiliated Hospital of Chongqing Medical University, Chongqing Key Laboratory of Ophthalmology and Chongqing Eye Institute, Chongqing, China; ^2^ Department of Ophthalmology, Yongchuan Hospital, Chongqing Medical University, Chongqing, China

**Keywords:** Behcet disease, VKH syndrome, TNFSF, TNFRSF

## Abstract

The present study was designed to explore the interrelationship between single nucleotide polymorphisms (SNP) of the tumor necrosis factor superfamily (TNFSF) and its respective receptor superfamily (TNFRSF) genes and Behcet's disease (BD) and Vogt-Koyanagi-Harada syndrome (VKH) in Han Chinese. The study sample included 796 patients with BD, 792 patients with VKH syndrome, and 1604 healthy controls. The genotyping of 35 SNPs was performed by MassARRAY platform (Sequenom), iPLEX Gold Assay, PCR-restriction fragment length polymorphism assay and TaqMan SNP assay. The mRNA expression levels of *TNFSF4, TNFSF8* and *TNFSF15* were analyzed by real-time PCR. The IL-6 and TNF-α expression levels were measured by ELISA. The A allele and AA genotype frequencies of *TNFSF4*/rs1234313 were significantly increased, and the GG genotype frequency of rs1234313 was decreased in subjects with BD. Significantly lower frequencies of the C allele and the CC genotype and higher frequencies of the TT and CT genotypes of *TNFSF15*/rs4246905 were observed in BD patients. A decreased frequency of the A allele of *TNFSF8*/rs7028891 was observed in BD patients. The expression of *TNFSF15* in CT carriers was significantly higher than that in CC/TT individuals. Increased IL-6 expression and TNF-α production were found in the *TNFSF15* CT carriers compared with the CC/TT genotype carriers. No significant differences were observed between the VKH patients and controls. This study indicates that *TNFSF4, TNFSF15* and *TNFSF8* may participate in the susceptibility to BD among Han Chinese.

## INTRODUCTION

Uveitis, also known as inflammation of the uvea, is a relatively common eye disease that occurs by an infectious or non-infectious mechanism and is one of the primary causes of visual impairment and blindness worldwide [1]. Behcet's disease (BD) and Vogt-Koyanagi-Harada disease (VKH) are the two main immune-mediated causes of uveitis and are relatively common in China [2, 3]. Notable differences exist in the pathogeneses and clinical features of these two entities. BD is a type of multi-systematic autoimmune disorder with an unclear etiology and pathogenesis, and the clinical manifestations include recurrent uveitis, oral aphthae, genital ulcers, erythema nodosum and a broad range of other syndromes [4, 5]. VKH entities are generally considered to be multisystem disorders characterized by ocular inflammation, neurological and dermatological manifestations and typical ocular features that always include chronic bilateral, non-necrotizing, granulomatous panuveitis with exudative retinal detachment [6]. Although the causes of uveitis remain unknown, genetic variants of immune-related genes are thought to contribute to the mechanism of the disease based on evidence of strong relationships with immune genes, including HLA genes and various non-HLA genes, such as *IL-37*, *IL-18RAP*, *ATG5*, *ATG10*, *FAS* and *TRAF5* [7–10].

The immune system is always in a state of equilibrium. The strict control of the proliferation and survival of immune cells in all phases of the immune response contributes to the establishment of immune system homeostasis. The tumor necrosis factor superfamily (TNFSF) and its respective receptor superfamily (TNFRSF) play critical roles in immune homeostasis, cell death and inflammation [11]. The TNFSF activated by the structural properties and the relative expression pattern of this superfamily [12]. These signaling pathways regulate cell survival, proliferation, differentiation, apoptosis and the effect or function of immune cells, which effectively assist in retaining immune cell homeostasis and in regulating the pathology of autoimmune diseases [13–15]. Interactions between TNFSF ligands and TNFRSF receptors comprise the initial process during inflammation, and the dysregulated or activated members of these superfamilies have been shown to be related to several immune-mediated diseases, such as systemic lupus erythematosus (SLE), multiple sclerosis (MS), inflammatory bowel disease (IBD), rheumatoid arthritis (RA), ankylosing spondylitis (AS), and Crohn's disease (CD) [16–20]; however, little is known about the effects of these interactions on the pathogenesis of uveitis. Additionally, one report indicated that the *TNFSF4* gene, one of the TNFSFs, may be related to BD and VKH [21].

We performed a study to investigate the associations of genetic polymorphisms in the genes of the two families with BD and VKH. Thirty-five single-nucleotide polymorphisms (SNPs) were selected based on earlier disease association studies. A case-control research design was implemented to investigate whether TNFSF and TNFRSF genes contributed to the susceptibilities to BD and VKH. The results indicated that *TNFSF4*/rs1234313, *TNFSF15*/rs4246905, and *TNFSF8*/rs7028891 were related to BD but not VKH. Functional studies indicated that TNFSF15/rs4246905 is likely involved in the development of BD via the upregulated production of inflammatory cytokines, e.g., IL-6 and TNF-α.

## RESULTS

### Clinical features

The clinical characteristics of BD and VKH patients are summarized in Table 1. The allele and genotype frequency distributions of the 35 SNPs in the healthy controls did not deviate from the Hardy-Weinberg equilibrium (*P* > 0.05).

**Table 1:** Clinical characteristics, sex, and age of BD and VKH patients with uveitis

	Total	%
**Parents with BD**	796	–
Mean age ± SD	33.7 ± 9.0	–
Male	662	83.2
Female	134	16.8
Uveitis	796	100
Oral ulcer	780	98
Genital ulcer	479	60.2
Skin lesions	624	78.4
Positive pathergy test	126	15.8
Arthritis	169	21.2
**Patients with VKH**	792	–
Mean age ± SD	39.5 ± 13.8	–
Male	429	54.2
Female	363	45.8
Uveitis	792	100
Headache	322	40.7
Tinnitus	363	45.8
Alopecia	327	41.3
Vitiligo	153	19.3
Poliosis	305	38.5
Healthy controls	1604	–
Mean age ± SD	39.6 ± 10.6	–
Male	898	56
Female	706	44

### Comparisons of the allele and genotype frequencies of the tested SNPs between the cases and controls in the first-phase study

A total of 35 SNPs of 412 BD patients, 408 VKH patients and 644 healthy individuals were genotyped in the first set of experiments. The results indicated significant differences between the BD patients and the controls in three SNPs, i.e., *TNFSF4*/rs1234313, *TNFSF15*/rs4246905, and *TNFSF8*/rs7028891. A significant decrease was observed in the GG genotype frequency of *TNFSF4*/rs1234313 in the BD patients compared to the controls (Pc = 0.013, OR = 0.513), whereas the A allele of *TNFSF*4/rs1234313 was markedly increased (Pc = 0.012, OR=1.421). The frequencies of the *TNFSF15*/rs4246905 C allele and CC genotype were obviously lower in the BD patients than those in the controls (Pc = 0.0024, OR = 0.670; and Pc = 0.0012, OR = 0.554, respectively). Regarding *TNFSF8*/rs7028891, a decreased frequency of the A allele was found in the BD patients compared to that in the healthy controls (Pc = 0.0086, OR = 0.664). A significant difference was also observed between the VKH patients and healthy individuals: the frequency of the *TNFSF13B*/rs9514828 C allele was significantly lower (Pc = 0.013, OR = 0.690) in the VKH patients (Supplementary Table 1).

### Comparisons of the allele and genotype frequencies of the tested SNPs between the cases and controls in the replication and combined studies

To confirm the possible associations of rs1234313, rs4246905, and rs7028891 with BD and rs9514828 with VKH, an additional set of 384 BD patients, 384 VKH patients and 960 healthy controls were enrolled. The replication results revealed that the A allele of *TNFSF4*/rs1234313 was markedly increased in the BD patients (Pc = 0.018, OR = 1.402). The frequencies of the *TNFSF15*/rs4246905 C allele and CC genotype were significantly lower in the BD patients (Pc = 1.37×10^−5^, OR = 0.633; and Pc = 7.50×10^−4^, OR = 0.568, respectively). Regarding *TNFSF8*/rs7028891, a decreased frequency of the A allele was found in BD patients (Pc = 0.028, OR = 0.736) compared to healthy controls. No significant association was observed between rs9514828 and the VKH patients in the replication study (Supplementary Table 1).

The data collected from the two stages indicated significant increases in the A allele and AA genotype frequency of *TNFSF4*/rs1234313 in BD patients (Pc = 4.21×10^−4^, OR = 1.342; and Pc = 0.044, OR = 1.374, respectively) compared to healthy controls, while the GG genotype frequency of *TNFSF4*/rs1234313 was decreased (Pc = 0.011, OR = 0.603). The frequencies of the *TNFSF15*/rs4246905 C allele and CC genotype were significantly lower in BD patients (Pc = 4.10 × 10^−9^, OR = 0.655; and Pc = 5.19 × 10^−8^, OR = 0.566, respectively), while the TT genotype frequency of *TNFSF15*/rs4246905 was increased (Pc = 0.008, OR = 1.639). Regarding *TNFSF8*/rs7028891, a decreased frequency of the A allele was found in BD patients (Pc = 0.019, OR = 0.788) compared to healthy controls. For *TNFSF13B*/rs9514828, no significant associations were observed for VKH patients (Supplementary Table 1).

### Influence of TNFSF15/rs4246905 genotypes on mRNA and cytokine expression

**Figure 1:**
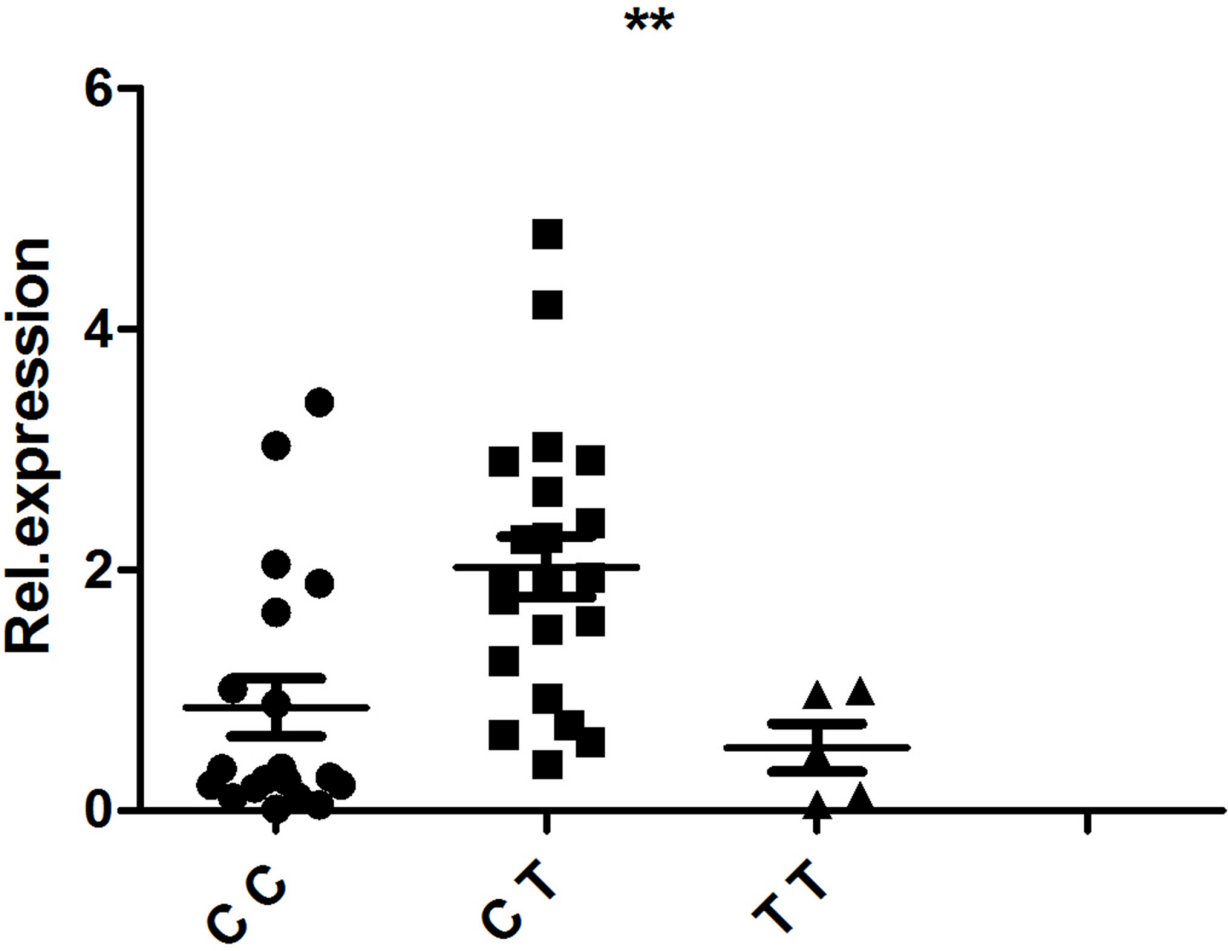
The influence of TNFSF15/rs4246905 genotypes (CC: *N* = 21, CT: *N* = 19, TT: *N* = 5) on the mRNA expression of TNFSF15 by PBMCs obtained from healthy genotyped individuals The expression of TNFSF15 in CT carriers was significantly higher than that in CC/TT individuals. ^**^P < 0.01.

**Figure 2:**
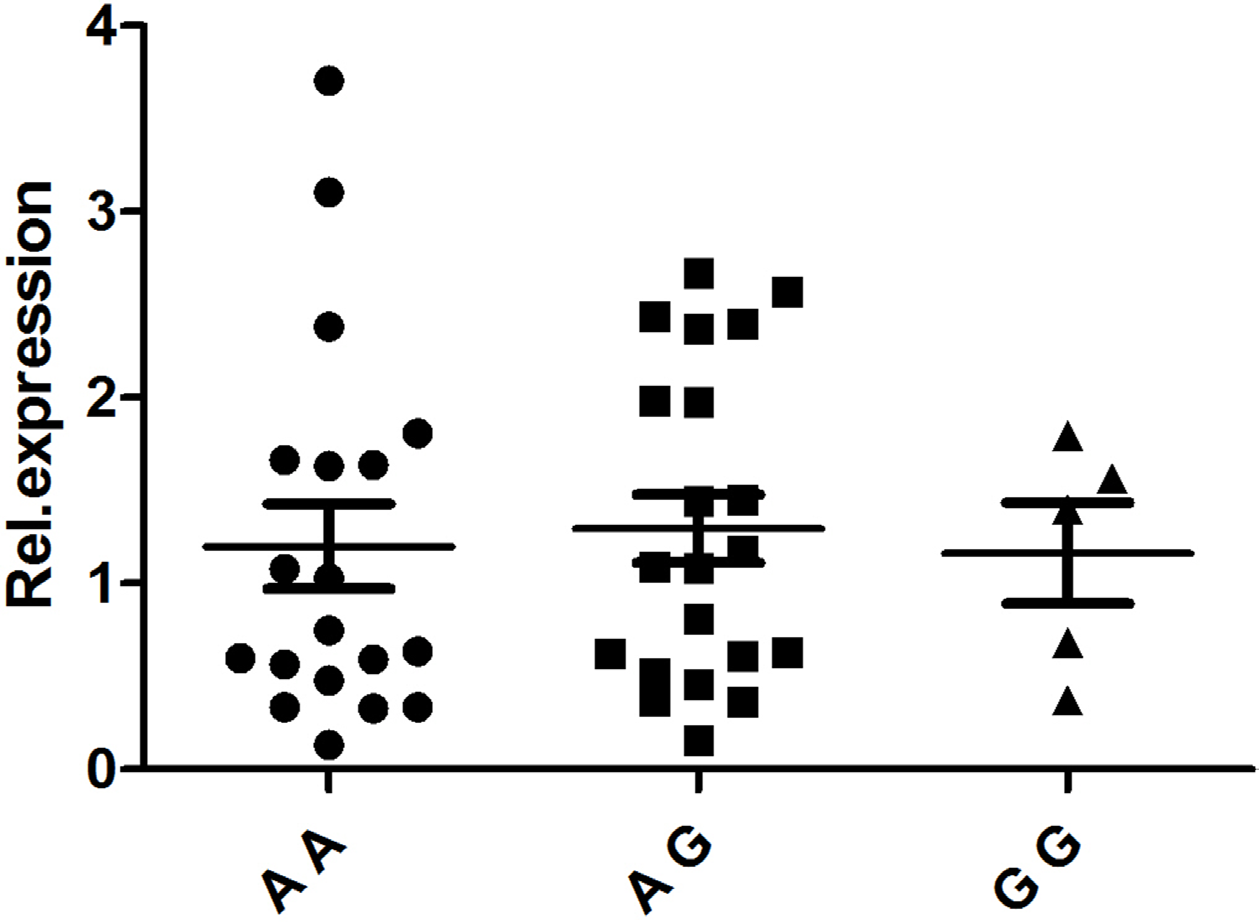
The influence of TNFSF4/rs1234313 genotypes (AA: *N* = 19, AG: *N* = 21, GG: *N* = 5) on the mRNA expression ofTNFSF4 in PBMCs The different genotypes of rs1234313 did not significantly affect TNFSF4 expression in PBMCs from healthy individuals.

**Figure 3:**
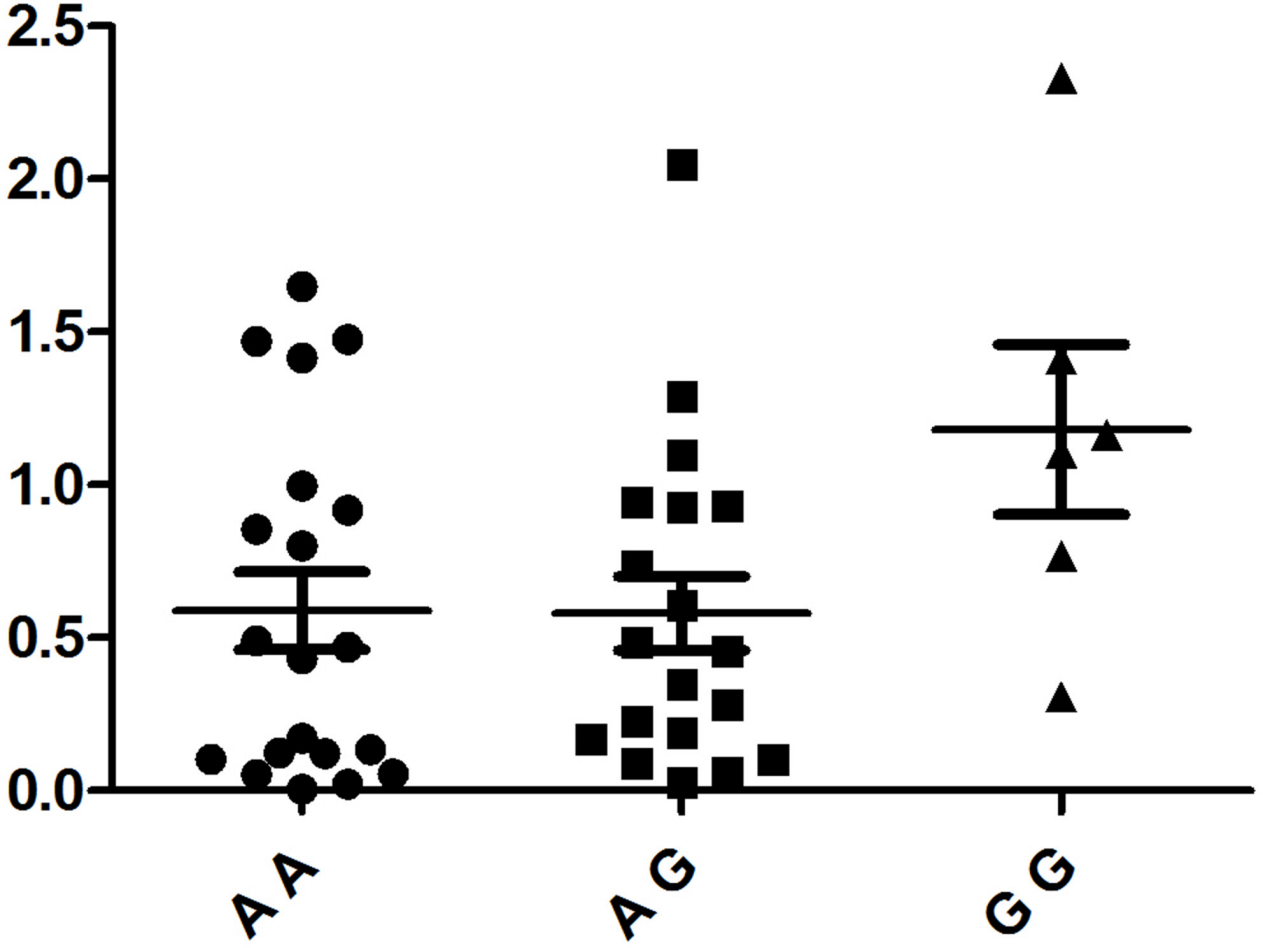
The influence of TNFSF8/rs7028891 genotypes (AA: *N* =20, AG: *N* = 19, GG: *N* = 6) on the mRNA expression ofTNFSF8 in PBMCs No statistically significant difference concerning TNFSF8 mRNA expression was detected between three genotypes.

PBMCs isolated from 48 healthy, genotyped individuals were used to explore the influences of different genotypes on mRNA and cytokine expression. Real-time PCR was used to determine whether the rs1234313, rs4246905 and rs7028891 polymorphisms altered the mRNA expression levels of the *TNFSF4, TNFSF15,* and *TNFSF8* genes. The results indicated that, compared to the mRNA expression of *TNFSF15*/rs4246905 in CC and TT carriers, the expression level in CT carriers was significantly increased (Figure 1). The different genotypes of rs1234313 and rs7028891 did not significantly affect *TNFSF4* and *TNFSF8* expression in PBMCs from healthy individuals (Figures 2 and 3).

**Figure 4:**
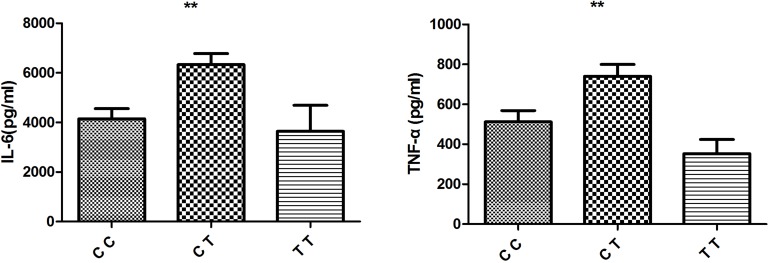
Effect of TNFSF15/rs4246905 genotype on cytokine production by LPS stimulated PBMCs from healthy genotyped individuals (CC: *N* = 18, CT = 20, and TT: *N* = 5) Data are expressed as the mean ± SD. ^**^P < 0.01.

According to these results, different genotypes of rs4246905 could affect *TNFSF15* expression following LPS stimulation, and further functional studies were performed to investigate whether the different genotypes of rs4246905 could also affect cytokine production. ELISAs were used to examine the IL-6 and TNF-α levels in cell culture supernatants following LPS stimulation of PBMCs from 48 healthy, genotyped individuals. The results indicated that IL-6 and TNF-α production by the stimulated PBMCs from the CT carriers of TNFSF15 was greater than that of the CC/TT carriers (Figure 4).

## DISCUSSION

This study indicated that three SNPs, i.e., *TNFSF4*/rs1234313, *TNFSF15*/rs4246905 and *TNFSF8*/rs7028891, were associated with BD but not with VKH in Han Chinese populations. *TNFSF13B*/rs9514828 was associated with VKH in the first-stage study, but no association was found in the replication or combined studies. This discrepancy could have been caused by the small sample size of the first-stage study. No association was found between the remaining members of the TNFSF and TNFRSF and BD or VKH. The functional experiments implied that, compared to the relative mRNA expression level of *TNFSF15*/rs4246905 among CC/TT carriers, the expression level among CT carriers was increased and that, compared to PBMCs from the other two genotypes, the PBMCs from the CT carriers exhibited significant increases in IL-6 and TNF-α production. Only one report has addressed the association of *TNFSF4* (a member of the TNFSF) with the two main causes of uveitis, and thus little is known about the associations of other TNFSF and TNFRSF SNPs with the two diseases.

The fact that no common associations were found between BD and VKH indicates that different immunological pathways are involved in the two main causes of uveitis. BD is associated with non-granulomatous inflammation and is caused by an aberrant inflammatory response to specific environmental triggers [22], whereas VKH is considered to be associated with granulomatous inflammation [23]. However, the pathologies of both diseases have been demonstrated to be associated with immune imbalances through T cells. CD4^+^ T cells play an important role in homeostasis, and according to previous reports, a crucial imbalance in T lymphocyte function can elicit immune or inflammatory disorders [24, 25]. CD4^+^ T cells are critical for host defense, but in addition to their key role as helper cells within the immune system, these cells can also be troublesome in that they can drive autoimmune diseases, e.g., IBD, MS and allergies [26]. Previous studies have demonstrated that pathogenic CD4^+^ T cells may elicit damaging effects that are involved in the processes of BD and VKH initiation and development, and the mechanism is likely related to the abnormal production of Th1, Th2, Th17 and Treg cytokines and the abnormal frequencies of these four T-cell subsets [27–31].

Most TNFSF members that are expressed by the cells of the immune system are essential for maintaining the balance of T cell-mediated immune responses by providing the effector and regulatory of T cells with straightforward signals that modulate the contraction and expansion of the T cell effector pool and the survival of memory T cells [32, 33]. Recent studies indicate that several TNFSF members, notably TNF (*TNFSF2*, also known as TNF-*α*) [34], FasL (*TNFSF6*, also known as CD95L) [35] and TRAIL (TNF-related apoptosis inducing ligand, *TNFSF10*) [36], contribute to the pathogenesis of certain autoimmune diseases, such as IBD and CD, by enhancing the proinflammatory functions of T cells. Such enhancements could be the reason that the three genes identified in this study contributed to the susceptibility to BD.

*TNFSF8*, also known as *CD30L*, could be capable of interacting with its receptor on effector or memory T helper cells by activating CD4^+^T cells, antigen-presenting cells, and neutrophils. This process is thought to be involved in the pathogeneses of certain inflammatory diseases, e.g., IBD, RA and CD [37, 38, 39]. In this study, *TNFSF8*/rs7028891 was associated with susceptibility to BD (Pc = 0.019). Recent research has demonstrated that signaling of *TNFSF8* and its receptor play key roles in the *in vitro* and *in vivo* differentiation of Th17 cells [40]. Interestingly, several lines of published evidence have indicated that Th17 cells may participate in the mechanism of uveitis [29, 31].

*TNFSF4*, which is located at 1q25.1, could be capable of encoding a cytokine that regulates T cells through CD28-independent costimulatory signals [41]. Moreover, the signal mediated by *TNFSF4* has been found to inhibit the function of IL-10-producing CD4^+^ type 1 regulatory T cells and the *in vitro* production of IL-17 [42]. Accumulating data suggest that the interaction between *TNFSF4* and its receptor, *TNFRSF4,* not only plays crucial roles in the induction of anti-tumor immunity, allergies and autoimmunity, but also suppresses the development of adaptive T regulatory (TR1) cells [43]. Genetic variations at *TNFSF4* have been reported to be associated with autoimmune disorders, such as SLE and certain inflammatory conditions, including atherosclerosis and RA. *TNFSF4*/rs1234313 was found to be associated with BD in this study (Pc = 4.21 × 10^−4^). Moreover, in another study, *TNFSF4*/rs1234315 was found to be involved in the susceptibility to BD (Pc = 1.44 × 10^−5^) [21]. Additionally, previous studies have indicated that blocking *TNFSF4* can inhibit ocular inflammation in a mouse model of experimental autoimmune uveitis, and activation of the *TNFSF4* receptor augments Th17 cell function and thereby contributes to ocular inflammation [44].

*TNFSF15* is located on human chromosome 9q33 and could encode tumor necrosis factor-like ligand 1a (TL1A), which is in a position to interact with death receptor 3 (DR3). These proteins could modulate the functions of T, NK, and NKT cells and thus drive the inflammatory reaction, affect all of the main effector pathways and induce the mucosal upregulation of the Th1, Th2, and Th17 factors, for example, in several T-cell-dependent autoimmune diseases [45, 46]. According to previous studies, the TL1A/DR3 pathway is involved in several diseases, including RA, CD, BD, SLE and AS. Significant increases in the systemic levels of TLA1 have been found in these related diseases. RA patients exhibit a decrease in serum TL1A levels following treatment with TNF-α-blocking antibodies [47]. Increases in the expression of DR3 in peripheral T lymphocytes and intestinal tissue have been discovered in IBD [48]. The plasma levels of TL1A were obviously higher in newly diagnosed SLE patients than in a control group [49], and the frequency of duplication of the DR3 gene is increased in RA patients compared to controls [50]. The fact that the polymorphisms of *TNFSF15* contribute to the susceptibilities to certain diseases, such as CD, further corroborates the roles of TL1A and DR3 in autoinflammatory disease. Additionally, studies have indicated that the genetic variants of *TNFSF15* might be implicated in the pathogenesis of acute anterior uveitis among Han Chinese individuals [51]. These findings show that the TL1A/DR3 pathway might participate in the pathological mechanism of uveitis.

A genome-wide association study that investigated differences in the genetic characteristics of European and Japanese IBD patients found that *TNFSF15*/rs4246905 is associated with the susceptibilities to CD (*P* = 7.25 × 10^−28^) and IBD (*P* = 1. 80 × 10^−21^) in a Japanese population [52]. Other studies have reported that the rs4246905 SNP is associated with ulcerative colitis (*P* = 6 × 10^−12^) [53] and IBD (*P* = 3 × 10^−32^) [54]. In this study, *TNFSF15*/rs4246905 was associated with the susceptibility to BD (Pc = 4.10 × 10^−9^). Together, these results indicate that the *TNFSF15*/rs4246905 gene polymorphism might be a risk factor for various diseases including CD, IBD, and BD.

Interestingly, the three SNPs that were found to be associated with BD in the present study all belonged to the TNFSF and not the TNFRSF. Variations in TNFSF genes occur more frequently in various types of autoimmune diseases, such as SLE, IBD, CD and others. This information may provide some evidence that variations in the TNFSF regions also play vital roles in the causes of this autoimmune disease; this possibility emphasizes the importance of the hypothesis that the genetic backgrounds of several autoimmune disorders overlap at least partially [55]. Regarding the TNFRSF, further study is needed to confirm its association with autoimmune diseases.

There are several limitations to our study. First, this research was conducted in a population limited to the Han majority in China; thus, further studies need to be conducted among other ethnic groups. Second, only two types of uveitis entities were examined in this survey; thus, our results cannot be validated for uveitis with other pathogeneses. Additionally, it is possible that other genes of the TNFSF and TNFRSF groups may also participate in the pathogenesis of BD because only a portion of the relevant genes were investigated in this survey. Furthermore, further research is necessary to determine the roles that different mutation variants play in the pathogenesis of BD and to identify efficient methods to treat this disease.

In summary, this study determined that *TNFSF4*/rs1234313, *TNFSF15*/rs4246905, and *TNFSF8*/rs7028891 were related to increases in the risk of BD among Han Chinese. This study further enhanced our knowledge of the costimulatory molecule-mediated immunopathological mechanisms of uveitis and supported the pivotal role of the TNFSF pathway in the mechanisms of diseases in which T cells play an important role. Finally, our results might make T cells attractive candidates for clinical interventions involving several types of biologic targeting.

## MATERIALS AND METHODS

### Study population

A total of 796 BD cases, 792 VKH cases and 1604 healthy controls were included in this research. The study was performed in two stages. In the first stage, 412 BD cases, 408 VKH cases and 644 healthy controls from a Han Chinese population who were age-, ethnicity- and geography-matched were recruited. The second stage comprised another 384 BD cases, 384 VKH cases and 960 age-matched controls. All participants were recruited from the First Affiliated Hospital of Chongqing Medical University (Chongqing, China) between October 2009 and July 2016. The diagnostic criteria for the two main forms of uveitis strictly followed the respective International Workshop criteria [56, 57]. Each of the participants provided written informed consent before blood collection.

### SNP selection and genotyping

Based on the results of previous research, the following 38 SNPs of 11 genes were selected as candidate SNPs for this study: *TNFSF4* (rs704840, rs1234315, rs10798269, rs1234302, rs1234313, rs844644, rs10489265, rs1012507, rs4916319, and rs3850641); *TNFSF8* (rs3181374, rs7863183, rs7028891, rs1322055, and rs3181362); *TNFSF13* (rs11552708 and rs3803800); *TNFSF13B* (rs9514828); *TNFSF15* (rs6478106, rs10817669, rs3810936, rs6478108, rs10759734, rs11554257, rs4246905, rs4979462, and rs7865494); *TNFRSF1A* (rs1800693, rs4149577, rs4149570, rs767455, and rs2234649); *TNFRSF1B* (rs1061622); *TNFRSF6B* (rs4809330 and rs2315008); *TNFRSF11A* (rs4263037); *TNFRSF11* (rs3102735); and *TNFRSF14* (rs6684865). Considering that 3 SNPs of *TNFSF4* (rs1234135, rs704840, and rs844644) have previously been studied, 35 SNPs were selected to investigate the roles of TNFSF and TNFRSF genes in the genetic predispositions to BD and VKH.

Genomic DNA from BD and VKH patients and controls was extracted from peripheral blood samples using QIAamp DNA Blood Mini Kits (QIAGEN, CA) in accordance with the manufacturer's protocols, checked for quality and concentration, and maintained at −20°C until use. The genotypes of 29 selected SNPs were identified with an iPLEX Gold Genotyping Assay (Applied Biosystems, Foster City, CA) and the MassARRAY platform (Sequenom Inc, San Diego, California, USA). The PCR protocol and detection primers for the candidate SNPs were designed by the MassArray Designer of Sequenom. TYPER software version 4.0 was used to analyze the experimental data. Genotyping of the remaining 6 SNPs was completed with TaqMan SNP assays or restriction fragment length polymorphism PCR. The genotyping success rate for all SNPs tested in this study ranged from 95.5% to 100%. To control the data quality prior to data analysis, measurements of the validity of the genotyping were performed using direct sequencing for 3% of the samples (Sangon Biotech, Shanghai, China).

### Real-time PCR analysis

PBMCs were isolated from the venous blood of 48 healthy controls using Ficoll-Hypaque density gradient centrifugation. Genomic RNA was extracted from the PBMCs with TRIzol (Invitrogen, Carlsbad, CA, USA), and reverse transcription was conducted with a transcriptase kit (TaKaRa, Japan) according to the manufacturer's instructions. The expression was analyzed with an ABI 7500 Real-Time PCR System with the following primers: *TNFSF15* (forward: 5′-GCTTCCTATCCTGGGAGACC-3′ and reverse: 5′-TGCTCAGGAGCCTCTCAAAT-3′); *TNFSF4* (forward: 5′-GGTATCACATCGGTATCCTCGA-3′ and reverse: 5′-TGAGTTGTTCTGCACCTTCATG-3′); *TNFSF8* (forward: 5′-CCAAGAAGTCATGGGCC TACCTCCAA-3′ and reverse: 5′-GCAAACGATG AAGTACAAGCCAGGGAA-3′); and β-actin (forward: 5′-CGAGAAGATGACCCAGATCATG-3′ and reverse: 5′-CAGAGGCGTACAGGGATAGCA-3′). The relative expression levels of *TNFSF15, TNFSF4,* and *TNFSF8* were normalized to the total level of β-actin expression and calculated via the 2^−ΔΔ Ct^ method.

### Detection of cytokine levels by ELISA

The cell culture supernatants of the PBMCs were stimulated with LPS (100 ng/ml; Sigma-Aldrich, USA) for 72 hours and collected and stored at −80°C until use. The concentrations of cytokines (IL-6 and TNF-α) were detected with Human Duoset ELISA kits (R&D Systems) according to the manufacturer's protocols. A standard curve was produced for each plate, and the absorbance (A) was read at 450 nm.

### Statistical analysis

Deviations from Hardy-Weinberg equilibrium were analyzed with chi-square (χ^2^) tests. The χ^2^ test was used to compare the allele and genotype frequencies of the tested SNPs between the cases and healthy controls using SPSS version 17.0. The risks were assessed as odds ratios (ORs) with 95% confidence intervals (CIs). The Bonferroni method was used to correct for multiple comparisons, and Pc values less than 0.05 were considered statistically significant. The non-parametric Mann-Whitney test was used to compare *TNFSF4*/rs1234313, *TNFSF15*/rs4246905 and *TNFSF8*/rs7028891 expression levels and the cytokine levels among the three genotype groups.

## SUPPLEMENTARY MATERIALS TABLE




