# Supplementary material for: Associations between TNFSF4, TNFSF8 and TNFSF15 and Behçet's disease but not VKH syndrome in Han Chinese
Source: Oncotarget. 2017 Oct 23;8(62):105037–46. doi: 10.18632/oncotarget.22064 (PMC5739618; doi:10.18632/oncotarget.22064)
Supplement: Supplementary file 1 [file oncotarget-08-105037-s001.pdf]

Associations between *TNFSF4, TNFSF8* and *TNFSF15* and Behçet's disease but not VKH syndrome in Han Chinese

SUPPLEMENTARY MATERIALS

Supplementary Table 1: Genotype and allele frequencies of TNFSF4/rs1234313, TNFSF15/rs4246905 and TNFSF8/rs7028891 polymorphisms in BD patients and healthy controls. See_Supplementary_Table_1
