# Supplementary material for: Associations between TNFSF4, TNFSF8 and TNFSF15 and Behçet's disease but not VKH syndrome in Han Chinese
Source: Oncotarget. 2017 Oct 23;8(62):105037–46. doi: 10.18632/oncotarget.22064 (PMC5739618; doi:10.18632/oncotarget.22064)
Supplement: Supplementary file 2 [file oncotarget-08-105037-s002.docx]

| **Supplementary Table 1: Genotype and allele frequencies of *TNFSF4*/rs1234313, *TNFSF15*/rs4246905 and *TNFSF8*/rs7028891 polymorphisms in BD patients and healthy controls** | | | | | | | | |
| --- | --- | --- | --- | --- | --- | --- | --- | --- |
| Gene | SNPs |  | GenotypeAllele | BD n(%) | Control | P value | Pc value | OR(95%CI) |
| TNFSF4 | rs1234313 | first | A | 476(61.3) | 631(52.8) | 1.758×10^-4^ | 0.012 | 1.421(1.182-1.707) |
|  |  |  | G | 300(38.7) | 565(47.2) |  |  | 0.704(0.586-0.846) |
|  |  |  | AA | 141(36.3) | 174(29.1) | 0.017 | NS | 1.391(0.060-1.826) |
|  |  |  | AG | 194(50.0) | 283(47.3) | 0.411 | NS | 1.113(0.862-1.437) |
|  |  |  | GG | 53(13.7) | 141(23.6) | 1.295×10^-4^ | 0.013 | 0.513(0.363-0.725) |
|  |  | second | A | 541(70.4) | 1180(63.0) | 2.51×10^-4^ | 0.018 | 1.402(1.169-1.680) |
|  |  |  | G | 227(29.6) | 694(37.0) |  |  | 0.713(0.595-0.855) |
|  |  |  | AA | 192(50.0) | 372(39.7) | 0.001 | NS | 1.519(1.196-1.929) |
|  |  |  | AG | 157(40.9) | 436(46.5) | 0.061 | NS | 0.795(0.625-1.011) |
|  |  |  | GG | 35(9.1) | 129(13.8) | 0.02 | NS | 0.628(0.424-0.932) |
|  |  | combined | A | 1017(65.9) | 1811(59.0) | 6.02×10^-6^ | 4.21×10^-4^ | 1.342(1.181-1.524) |
|  |  |  | G | 527(34.1) | 1259(41.0) |  |  | 0.745(0.656-0.847) |
|  |  |  | AA | 333(43.1) | 546(35.6) | 4.15×10^-4^ | 0.044 | 1.374(1.152-1.639) |
|  |  |  | AG | 351(45.5) | 719(46.8) | 0.532 | NS | 0.946(0.795-1.126) |
|  |  |  | GG | 88(11.4) | 270(17.6) | 1.07×10^-4^ | 0.011 | 0.603(0.466-0.780) |
| TNFSF15 | rs4246905 | first | C | 459(59.8) | 809(68.9) | 3.49×10^-5^ | 2.4×10^-3^ | 0.670(0.554-0.810) |
|  |  |  | T | 309(40.2) | 365(31.1) |  |  | 1.492(1.234-1.804) |
|  |  |  | CC | 133(34.6) | 287(48.9) | 1.16×10^-5^ | 1.22×10^-3^ | 0.554(0.425-0.722) |
|  |  |  | CT | 193(50.3) | 235(40.0) | 0.002 | NS | 1.514(1.168-1.962) |
|  |  |  | TT | 58(15.1) | 65(11.1) | 0.065 | NS | 1.429(0.977-2.089) |
|  |  | second | C | 438(57.2) | 1270(67.8) | 1.96×10^-7^ | 1.37×10^-5^ | 0.633(0.532-0.752) |
|  |  |  | T | 328(42.8) | 602(32.2) |  |  | 1.580(1.329-1.878) |
|  |  |  | CC | 128(33.4) | 439(46.9) | 7.14×10^-6^ | 7.50×10^-4^ | 0.568(0.443-0.728) |
|  |  |  | CT | 182(47.5) | 392(41.9) | 0.061 | NS | 1.257(0.990-1.596) |
|  |  |  | TT | 73(19.1) | 105(11.2) | 1.543×10^-4^ | 0.016 | 1.864(1.345-2.582) |
|  |  | combined | C | 897(58.5) | 2079(68.3) | 5.85×10^-11^ | 4.10×10^-9^ | 0.655(0.577-0.744) |
|  |  |  | T | 637(41.5) | 967(31.7) |  |  | 1.527(1.345-1.734) |
|  |  |  | CC | 261(34.0) | 726(47.7) | 4.94×10^-10^ | 5.19×10^-8^ | 0.566(0.473-0.678) |
|  |  |  | CT | 375(48.9) | 627(41.2) | 4.38×10^-4^ | 0.046 | 1.367(1.148-1.628) |
|  |  |  | TT | 131(17.1) | 170(11.1) | 7.64×10^-5^ | 8.02×10^-3^ | 1.639(1.281-2.098) |
| TNFSF8 | rs7028891 | first | A | 570(72.3) | 965(79.8) | 1.23×10^-4^ | 8.61×10^-3^ | 0.664(0.538-0.819) |
|  |  |  | G | 218(27.7) | 245(20.2) |  |  | 1.506(1.221-1.858) |
|  |  |  | AA | 203(51.5) | 378(62.5) | 0.001 | NS | 0.638(0.494-0.825) |
|  |  |  | AG | 164(41.6) | 209(34.5) | 0.024 | NS | 1.351(1.041-1.754) |
|  |  |  | GG | 27(6.9) | 18(3.0) | 0.004 | NS | 2.399(1.303-4.418) |
|  |  | second | A | 412(53.9) | 1147(61.4) | 3.963×10^-4^ | 0.028 | 0.736(0.621-0.872) |
|  |  |  | G | 352(46.1) | 721(38.6) |  |  | 1.359(1.147-1.611) |
|  |  |  | AA | 107(28.0) | 338(36.2) | 0.004 | NS | 0.686(0.529-0.890) |
|  |  |  | AG | 198(51.8) | 471(50.4) | 0.644 | NS | 1.058(0.834-1.342) |
|  |  |  | GG | 77(20.2) | 125(13.4) | 0.002 | NS | 1.634(1.195-2.234) |
|  |  | combined | A | 982(63.3) | 2112(68.6) | 2.675×10^-4^ | 0.019 | 0.788(0.693-0.896) |
|  |  |  | G | 570(36.7) | 966(31.4) |  |  | 1.269(1.116-1.443) |
|  |  |  | AA | 310(40.0) | 716(46.5) | 0.003 | NS | 0.765(0.642-0.911) |
|  |  |  | AG | 362(46.6) | 680(44.2) | 0.26 | NS | 1.105(0.929-1.313) |
|  |  |  | GG | 104(13.4) | 143(9.3) | 0.002 | NS | 1.511(1.155-1.977) |
| Gene | SNPs |  | GenotypeAllele | VKH n(%) | Control | P value | Pc value | OR(95%CI) |
| TNFSF13B | rs9514828 | first | C | 448(61.5) | 823(69.9) | 1.791×10^-4^ | 0.013 | 0.690(0.568-0.838) |
|  |  |  | T | 280(38.5) | 355(30.1) |  |  | 1.449(1.193-1.760) |
|  |  |  | CC | 144(39.6) | 291(49.4) | 0.003 | NS | 0.670(0.514-0.874) |
|  |  |  | CT | 160(44.0) | 241(40.9) | 0.356 | NS | 1.133(0.870-1.475) |
|  |  |  | TT | 60(16.4) | 57(9.7) | 0.002 | NS | 1.842(1.249-2.718) |
|  |  | second | C | 472(62.1) | 1187(64.7) | 0.206 | NS | 0.893(0.750-1.064) |
|  |  |  | T | 288(37.9) | 647(35.3) |  |  | 1.119(0.940-1.334) |
|  |  |  | CC | 139(36.6) | 379(41.3) | 0.112 | NS | 0.819(0.640-1.048) |
|  |  |  | CT | 194(51.1) | 429(46.8) | 0.161 | NS | 1.186(0.934-1.507) |
|  |  |  | TT | 47(12.3) | 109(11.9) | 0.808 | NS | 1.046(0.726-1.507) |
|  |  | combined | C | 920(61.8) | 2010(66.7) | 0.001 | NS | 0.807(0.710-0.919) |
|  |  |  | T | 568(38.2) | 1002(33.3) |  |  | 1.238(1.088-1.409) |
|  |  |  | CC | 283(38.0) | 670(44.5) | 0.004 | NS | 0.766(0.640-0.917) |
|  |  |  | CT | 354(47.6) | 670(44.5) | 0.166 | NS | 1.133(0.950-1.351) |
|  |  |  | TT | 107(14.4) | 166(11.0) | 0.022 | NS | 1.356(1.045-1.760) |
